# SUMOylation regulates USP5-Cav3.2 calcium channel interactions

**DOI:** 10.1186/s13041-019-0493-9

**Published:** 2019-08-27

**Authors:** Agustin Garcia-Caballero, Fang-Xiong Zhang, Lina Chen, Said M’Dahoma, Junting Huang, Gerald W. Zamponi

**Affiliations:** Department of Physiology and Pharmacology, Alberta Children’s Hospital Research Institute, Hotchkiss Brain Institute, Cumming School of Medicine, University of Calgary, Calgary, T2N 4N1 Canada

**Keywords:** T-type, Ubiquitination, SUMOylation, USP5, Cav3.2, Calcium channel, Pain

## Abstract

Cav3.2 calcium channels play a key role in nociceptive signaling in the primary afferent pain pathway. We have previously reported the regulation of Cav3.2 calcium channels by the deubiquitinase USP5 and its importance for regulating peripheral transmission of pain signals. Here we describe the regulation of the Cav3.2-USP5 interaction by SUMOylation. We show that endogenous USP5 protein expressed in dorsal root ganglia undergoes SUMOylation, and the level of USP5 SUMOylation is reduced following peripheral nerve injury. SUMO prediction software identified several putative lysines that have the propensity to be targets for SUMO conjugation. A series of single lysine substitutions in an mCherry tagged USP5 construct followed by expression in tsA-201 cells identified lysine K113 as a key target for USP5 SUMO2/3 modification. Finally, Cav3.2 calcium channel immunoprecipitates revealed a stronger interaction of Cav3.2 with a SUMO2/3 resistant USP5-K113R mutant, indicating that SUMO2/3 modification of USP5 reduces its affinity for the calcium channel Cav3.2. Collectively, our data suggest that dysregulation of USP5 SUMOylation after peripheral nerve injury may contribute to the well described alteration in Cav3.2 channel activity during neuropathic pain states.

## Introduction

Ubiquitination and deubiquitination are post-translational modifications that regulate protein stability through the modification of lysine residues by attachment/removal of one or more ubiquitin groups [1]. The mammalian genome expresses hundreds of ubiquitin ligases, and approximately one hundred deubiquitinases, thus underscoring the importance of ubiquitination for cell function [1, 2]. Among these, USP5 (or isopeptidase T) is a deubiquitinase that regulates different cell cycle modulators and has been shown to play important roles in various cancers [3–5]. In the nervous system, USP5 has been shown to be involved in the development of neuropathic and inflammatory pain states [6]. In rodent models of neuropathic, inflammatory, visceral and postsurgical pain, USP5 protein expression levels in dorsal root ganglia (DRG) and spinal cord are upregulated [6–10]**,** leading to an increased association with Cav3.2 T-type calcium channels and consequently their enhanced deubiquitination. This culminates in increased Cav3.2 channel density in these tissues, and hence a pronociceptive effect, whereas interfering with USP5 association with the channel mediates analgesia [6]. Although USP5 expression has been shown to be activity dependent [11], little is known about the cellular mechanisms that regulate USP5 association with Cav3.2.

Similar to ubiquitination, SUMO (SUMO; small ubiquitin-related modifier) conjugation (i.e., SUMOylation) involves the covalent attachment of SUMO (an 11 kDa peptide) to target proteins at specific lysine residues. SUMOylation is a reversible post-translational modification, but unlike ubiquitination, SUMOylation is not responsible for targeted protein degradation [12]. Instead SUMOylation of proteins such as transcription factors and protein kinases results in tight transcriptional regulation of specific genes involved in chromatin remodeling, cell cycle regulation, and subcellular trafficking amongst others [12]. SUMOylation also affects the regulation of Nav1.7 sodium channels by collapsin-response-mediator-protein 2 (CRMP2) and this in turn is highly important for the transmission of peripheral pain signals [13–17].

Here, we report the effects of SUMOylation on USP5 interactions with Cav3.2 calcium channels. We show that SUMOylation of USP5 is dysregulated after peripheral never injury, and show that a SUMO-deficient USP5 mutant displays increased interactions with Cav3.2 calcium channels. Our data suggest that SUMOylation has the propensity to modulate nociceptive signaling mediated by Cav3.2 channels.

## Methods

### Cell culture and transfection

Human embryonic kidney cells (tsA-201) were cultured as described by us previously [18]**.** Cells were transfected with lipofectamine 2000 and used for biochemical analysis 48–72 h post-transfection. Harvesting of DRG (L4-L6) from sham and SNI mice was performed as described previously [6].

### Site directed mutagenesis and plasmids

The open reading frame of the *Homo sapiens* ubiquitin specific peptidase 5 (USP5) (Gene symbol: USP5, GenBank entry: NM_001098536) was cloned into a pcDNA3.1 vector. To generate mCherry tagged USP5 (mCherry-USP5), the coding sequence of mCherry was amplified by PCR with the stop codon removed and inserted upstream of USP5. PCR was used to generate mutants of USP5 (K27R, K80R, K113R, K163R, K247R, K574R, K824R). All DNA constructs were confirmed by DNA sequencing. To generate the Cav3.2-GFP-tagged plasmid, the coding sequence of human Cav3.2 was cloned into the pcDNA3.1(+) vector (Invitrogen) with the stop codon removed; GFP was amplified by PCR and attached to the C-terminus of Cav3.2.

### Co-immunoprecipitation assays

tsA-201 cells or DRG tissues were lysed in a modified RIPA buffer (in mM; 50 Tris, 100 NaCl, 0.2% (v/v) Triton X-100, 0.2% (v/v) NP-40, 10 EDTA + protease inhibitor cocktail, pH 7.5) that was used to co-immunoprecipitate recombinant mCherry-USP5 with Cav3.2-GFP tagged channels with, SUMO2/3 with mCherry-USP5, or native SUMO2/3 with USP5. Lysates were prepared by sonicating samples at 60% pulse for 10 s and by centrifugation at 13,000 rpm for 15 min at 4 °C. Supernatants were transferred to new tubes and solubilized proteins were incubated with 50 μl of Protein G/A beads (Piercenet) and 2 μg of anti-GFP antibody (Abcam) overnight while tumbling at 4 °C. Total inputs were taken from whole cell samples representing 4% of total protein and probed for actin or α-Tubulin. Co-immunoprecipitates were washed twice with (mM) 150 NaCl 50 Tris pH 7.5 buffer, beads were aspirated to dryness. Laemmli buffer was added and samples were incubated at 96 °C for 7 min. Eluted samples were loaded on 7.5% or 10% Tris-glycine gel and resolved using SDS-PAGE. Samples were transferred to 0.45 mm polyvinylidenedifluoride (PDVF) membranes by dry transfer using an Iblot2 machine (Invitrogen).

### Western blots

Western blot assays were performed using anti-actin (Sigma) and anti-mCherry (Abcam) mouse antibodies, anti-α-Tubulin (Abcam), anti-GFP (Abcam), anti- SUMO 2/3 (Santa Cruz Biotechnology, Inc.), anti-USP5 (ProteinTech) rabbit antibodies. Western blot quantification was performed using densitometry analysis (Quantity One-BioRad software).

### SNI model

Surgeries for spared never injury were performed on 7–8 week old C57BL/6 J mice as previously described [19]. Briefly, a 0.5 cm incision was made on the skin of the left thigh under isoflurane anesthesia to expose the sciatic nerve. Tibial and common peroneal branches of the sciatic nerves were tightly ligated with a 6–0 silk suture (Ethicon, USA) and transected, leaving the sural nerve intact. A 1 mm piece of the ligated nerves was removed. The overlaying muscles and skin were closed with 6–0 silk and 4–0 vicryl sutures, respectively. For sham mice, surgeries were performed exactly as for SNI, but without nerve ligation and transection. Lumbar dorsal root ganglia (L4-L6) were harvested 2 weeks after surgeries.

### Statistical analysis

Data are presented as means and standard errors. Statistical analysis was performed using unpaired Student’s t-tests or One Way Analysis of Variance (ANOVA), with significance set at 0.05.

## Results and discussion

**Fig. 1:**
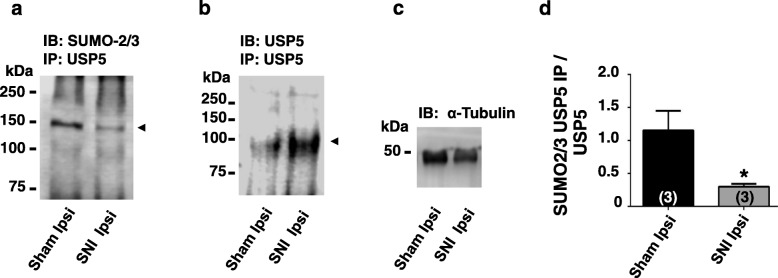
Endogenous USP5 SUMOylation levels in sham operated mice and in mice with a sciatic nerve injury. **a**. USP5 immunoprecipitates reveal that USP5 is SUMOylated, as seen by western blots probed against SUMO 2/3.Furthermore, there is a decrease in SUMO2/3 signal in ipsilateral DRGs from SNI mice when compared to ipsilateral DRGs from Sham mice, **b**. USP5 immunoprecipitates show increased USP5 levels in ipsilateral DRGs from SNI mice when compared to ipsilateral DRGs from Sham mice **c**. Protein control using an α-Tubulin antibody to probe DRG tissue from ipsilateral Sham or ipsilateral SNI mice. **d**. Densitometry analysis of USP5 SUMO 2/3 signals from USP5 immunoprecipitates normalized to USP5 expression levels. Numbers in parentheses reflect numbers of mice, the asterisk denotes statistical significance at the 0.05 level

We first examined whether USP5 endogenously expressed in DRG neurons is subject to SUMOylation. DRG neurons (L4-L6) were isolated from sham operated male wild type mice and USP5 immunoprecipitates were assayed by Western blotting. This experiment revealed that endogenously expressed USP5 is subject to SUMO conjugation (Fig. 1). We then compared USP5 SUMO levels between sham operated mice, and mice with a spared injury of the sciatic nerve. As shown in Fig. 1 a and c, we observed a three-fold decrease in USP5 SUMOylation following nerve injury, despite an overall injury-induced increase in USP5 protein levels as described earlier [6] (Fig. 1 b). These data suggest that USP5 SUMOylation is dynamically regulated during neuropathic pain states.

**Fig. 2:**
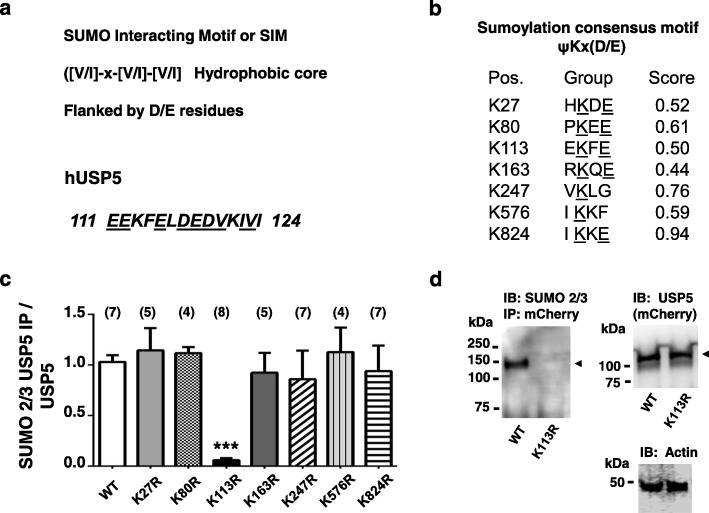
SUMO 2/3 conjugation of USP5 lysine 113 in tsA-201 cells. **a**. Depiction of the SUMO interacting motif (SIM) present in hUSP5 sequence. Note that a similar motif (EDKFEFDEDVKIVI) is present in mouse USP5. **b**. SUMOylation prediction score for hUSP5 lysines. **c**. Quantification of SUMOylated USP5 relative to total USP5 for mCherry tagged wild type USP5 and USP5 lysine mutants. The K113R single mutant abolishes USP5 SUMO 2/3 conjugation. This experiment was performed in tsA-201 cells, *n* = 5–8 transfections. Numbers in parentheses depict numbers of experiments. The asterisks indicate statistical significance relative to WT (ANOVA) at the 0.001 level. **d**. Representative blot for experiments such as those presented in panel c. USP5-K113R lacks SUMOylation type 2/3 as seen by western blot. USP5-WT and K113R mutant levels of expression as well as actin controls are shown

A SUMO interacting motif (SIM) is commonly present in proteins that undergo SUMO conjugation [20, 21], and typically comprises a valine / isoleucine hydrophobic core which is flanked by aspartic and/or glutamic residues. An analysis of human USP5 protein sequence reveals a motif between residues 111 and 124 that fulfills such a requirement and this motif is conserved in mouse USP5 (Fig.2 a). SUMO-modified proteins typically contain the tetrapeptide motif B-K-x-D/E where B is a hydrophobic residue, K is the lysine conjugated to SUMO, x is any amino acid, D or E is an acidic residue [22]. A scan for such motifs in USP5 with SUMO modification prediction software (https://www.abgent.com/sumoplot) revealed twelve lysine residues that fulfill such a requirement, and we centered our attention only to those with the 7 highest scores (Fig. 2 b**).** To determine which residues are responsible for USP5 SUMOylation, we first transfected wild type USP5 tagged with mCherry into tsA-201 cells, immunoprecipitated USP5 protein using an mCherry antibody, and then tested for SUMOylation using Western blot analysis (Fig. 1 c and d). These experiments revealed a robust SUMO signal in wild type USP5, consistent with our data obtained in native tissue. We then repeated a similar approach for single USP5 point mutants in which the seven lysines were individually substituted for arginine to maintain charge. These experiments revealed that SUMOylation was selectively abolished upon replacement of lysine 113 (Fig. 2 c and d) which lies within the SIM motif depicted in Fig. 2 a**,** altogether indicating that SUMOylation of USP5 occurs at a single locus. This lysine residue is also conserved in mouse USP5 (see legend to Fig. 2a).

**Fig. 3:**
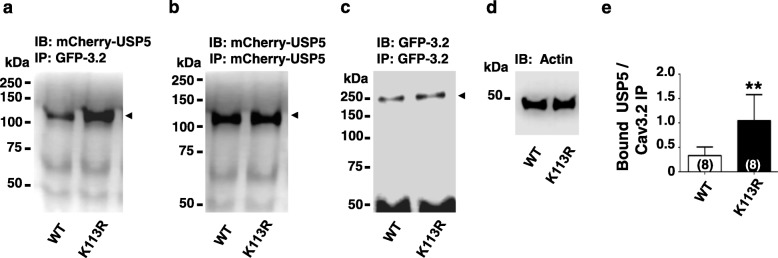
SUMO-resistant USP5-K113R shows enhanced Cav3.2 / USP5 interactions. **a**. Representative western blots of Cav3.2-GFP tagged immunoprecipitates showing increased binding to USP5-K113R mCherry tagged mutant. The blot was probed with an mCherry antibody. **b**.USP5-WT and the USP5-K113R mutant show equal levels of expression as detected by immunoprecipitation followed by western blot using an mCherry mouse antibody. **c**. Cav3.2-GFP tagged immunoprecipitates showed similar levels of expression when co-expressed with mCherry-tagged USP5-WT or mCherry tagged USP5-K113R mutant as detected by western blot using a GFP rabbit polyclonal antibody. **d**. Actin control using an actin antibody to probe whole cell lysates expressing either Cav3.2-GFP + USP5-WT (left lane) or Cav3.2-GFP + USP5-K113R (right lane). **e**. Densitometry analysis of USP5-WT or USP5-K113R bound to Cav3.2-GFP immunoprecipitates. Numbers in parentheses reflect numbers of separate experiments, asterisks denote statistical significance at the 0.01 level

We then sought to determine whether SUMOylation of USP5 affects the interaction between USP5 and Cav3.2 channels. As shown in Fig. 3**,** the USP5-K113R SUMO-resistant mutant more strongly (~ 2.2 fold) interacted with Cav3.2-GFP immunoprecipitates than USP5-WT (Fig. 3 a-e). This experiment suggests that SUMOylation state of USP5 can regulate USP5 interactions with Cav3.2 calcium channels. This in turn is expected to alter Cav3.2 stability via regulation of ubiquitination.

Overall, our observations extend our previous findings showing that peripheral nerve injury upregulates USP5 levels and leads to an enhanced interaction between USP5 and Cav3.2 [6]. Decreased SUMOylation of USP5 after nerve injury would aid this process such that USP5 interactions with Cav3.2 are enhanced when USP5 SUMOylation is decreased. Hence, preventing deSUMOylation of USP5 could provide a strategy for enhancing USP5 interactions with Cav3.2, which would in turn be predicted to lead to analgesia. We acknowledge the caveat that a USP5 construct with a point mutation that prevents SUMOylation is not strictly equivalent to deSUMOylation of USP5. It is therefore possible that the replacement of the lysine residue in position 113 with arginine could itself alter the three dimensional structure of USP5 and thus indirectly affect binding to Cav3.2, however, the notion that charge of residue 113 was preserved in our mutagenesis experiments minimizes this possibility. We have shown previously that the first half of the cUBP domain of USP5 is the key structural determinant of USP5 binding to Cav3.2 [8]. In contrast, residue 113 is located in the middle of the adjacent nUBP domain [23], suggesting that effects of lysine 113 SUMOylation on Cav3.2 interactions are unlikely due to steric hindrance, but instead occur allosterically.

At this point, we do not know if the alterations in USP5 SUMOylation following nerve injury are due to decreased activity of SUMO ligases, or an enhanced activity of SUMO proteases (SENPs). We also do not know which of the SUMO ligases and proteases are critical for regulating USP5 SUMOylation. It is interesting to note that a recent study has shown that the SUMO protease SENP1 is important for suppressing inflammatory pain, such that SENP1 null mice showed increased thermal hyperalgesia in models of inflammatory pain [24] – an effect that was attributed to an action on TRPV1 channels. As noted earlier, SUMOylation has also been implicated as an important factor in the modulation of Nav1.7 sodium channels by CRMP2, where prevention of CRMP2 SUMOylation [13, 15, 25] led to a reduction on whole cell Nav1.7 sodium currents in sensory neurons, and thus analgesia.

Overall, our data and recent findings by others implicate SUMOylation as an emerging mechanism for regulating signalling in the afferent pain pathway that may potentially be exploited towards development of new therapeutic strategies [26].

## Data Availability

The data used in our study are available from the authors on reasonable request.

## Abbreviations

CRMP2: Collapsin response mediator protein 2
DRG: Dorsal root ganglia
GFP: Green Fluorescent Protein
SNI: Sciatic Nerve Injury
SUMO: Small ubiquitin-related modifier

## References

[1] Komander D, Clague MJ, Urbe S (2009). Breaking the chains: structure and function of the deubiquitinases. Nat Rev Mol Cell Biol.

[2] Clague MJ, Barsukov I, Coulson JM, Liu H, Rigden DJ, Urbe S (2013). Deubiquitylases from genes to organism. Physiol Rev.

[3] Li XY, Wu HY, Mao XF, Jiang LX, Wang YX (2017). USP5 promotes tumorigenesis and progression of pancreatic cancer by stabilizing FoxM1 protein. Biochem Biophys Res Commun.

[4] Kaistha BP, Krattenmacher A, Fredebohm J (2017). The deubiquitinating enzyme USP5 promotes pancreatic cancer via modulating cell cycle regulators. Oncotarget.

[5] Ma X, Qi W, Pan H, Yang F, Deng J (2018). Overexpression of USP5 contributes to tumorigenesis in non-small cell lung cancer via the stabilization of beta-catenin protein. Am J Cancer Res.

[6] Garcia-Caballero A, Gadotti VM, Stemkowski P (2014). The deubiquitinating enzyme USP5 modulates neuropathic and inflammatory pain by enhancing Cav3.2 channel activity. Neuron.

[7] Gadotti VM, Caballero AG, Berger ND (2015). Small organic molecule disruptors of Cav3.2 - USP5 interactions reverse inflammatory and neuropathic pain. Mol Pain.

[8] Garcia-Caballero Agustin, Gadotti Vinicius M, Chen Lina, Zamponi Gerald W (2016). A cell-permeant peptide corresponding to the cUBP domain of USP5 reverses inflammatory and neuropathic pain. Molecular Pain.

[9] Joksimovic Sonja L., Joksimovic Srdjan M., Tesic Vesna, García-Caballero Agustin, Feseha Simon, Zamponi Gerald W., Jevtovic-Todorovic Vesna, Todorovic Slobodan M. (2018). Selective inhibition of CaV3.2 channels reverses hyperexcitability of peripheral nociceptors and alleviates postsurgical pain. Science Signaling.

[10] Gadotti VM, Zamponi GW (2018). Disrupting USP5/Cav3.2 interactions protects female mice from mechanical hypersensitivity during peripheral inflammation. Molecular brain.

[11] Stemkowski P, García-Caballero A, Gadotti VM, al e (2016). TRPV1 nociceptor activity initiates USP5/T-type channel-mediated plasticity. Cell Rep.

[12] Chang SC, Ding JL (2018). Ubiquitination and SUMOylation in the chronic inflammatory tumor microenvironment. Biochim Biophys Acta Rev Cancer.

[13] Francois-Moutal L, Dustrude ET, Wang Y (2018). Inhibition of the Ubc9 E2 SUMO-conjugating enzyme-CRMP2 interaction decreases NaV1.7 currents and reverses experimental neuropathic pain. Pain.

[14] Moutal A, Cai S, Luo S, Voisin R, Khanna R (2018). CRMP2 is necessary for Neurofibromatosis type 1 related pain. Channels.

[15] Moutal A, Dustrude ET, Largent-Milnes TM, Vanderah TW, Khanna M, Khanna R (2018). Blocking CRMP2 SUMOylation reverses neuropathic pain. Mol Psychiatry.

[16] Dustrude ET, Moutal A, Yang X, Wang Y, Khanna M, Khanna R (2016). Hierarchical CRMP2 posttranslational modifications control NaV1.7 function. Proc Natl Acad Sci U S A.

[17] François-Moutal L, Scott DD, Perez-Miller S, Gokhale V, Khanna M, Khanna R (2018). Chemical shift perturbation mapping of the Ubc9-CRMP2 interface identifies a pocket in CRMP2 amenable for allosteric modulation of Nav1.7 channels. Channels (Austin).

[18] Altier C, Garcia-Caballero A, Simms B (2011). The Cavbeta subunit prevents RFP2-mediated ubiquitination and proteasomal degradation of L-type channels. Nat Neurosci.

[19] Zhang Z, Gadotti VM, Chen L, Souza IA, Stemkowski PL, Zamponi GW (2015). Role of Prelimbic GABAergic circuits in sensory and emotional aspects of neuropathic pain. Cell Rep.

[20] Cong L, Pakala SB, Ohshiro K, Li DQ, Kumar R (2011). SUMOylation and SUMO-interacting motif (SIM) of metastasis tumor antigen 1 (MTA1) synergistically regulate its transcriptional repressor function. J Biol Chem.

[21] Kim ET, Kim KK, Matunis MJ, Ahn JH (2009). Enhanced SUMOylation of proteins containing a SUMO-interacting motif by SUMO-Ubc9 fusion. Biochem Biophys Res Commun.

[22] Chang CC, Tung CH, Chen CW, Tu CH, Chu YW (2018). SUMOgo: prediction of sumoylation sites on lysines by motif screening models and the effects of various post-translational modifications. Sci Rep.

[23] Avvakumov GV, Walker JR, Xue S (2012). Two ZnF-UBP domains in isopeptidase T (USP5). Biochemistry.

[24] Wang Y, Gao Y, Tian Q (2018). TRPV1 SUMOylation regulates nociceptive signaling in models of inflammatory pain. Nat Commun.

[25] Baumann A, Kursula P (2017). SUMO on CRMPs - wrestling for pain?. Channels.

[26] Moutal A, White KA, Chefdeville A, et al. Dysregulation of CRMP2 post-translational modifications drive its pathological functions. Mol Neurobiol. 2019. 10.1007/s12035-019-1568-4. 10.1007/s12035-019-1568-4 PMC6728212 30915713

